# Comprehensive assessment of homologous recombination deficiency via simultaneous methylation and mutation analysis in epithelial ovarian cancer: implications for PARP inhibitors efficacy

**DOI:** 10.1186/s40364-025-00843-6

**Published:** 2025-10-10

**Authors:** Lin Dong, Huanwen Wu, Ning Li, Wenbin Li, Yan Song, Yuanyuan Xiong, Huan Yin, Huan Fang, Rongrong Chen, Xin Yi, Jie Huang, Jianming Ying

**Affiliations:** 1 Department of Pathology, National Cancer Center, National Clinical Research Center for Cancer/Cancer Hospital, Chinese Academy of Medical Sciences and Peking Union Medical College, Beijing, 100021 China; 2 Department of Pathology, State Key Laboratory of Complex Severe and Rare Diseases, Molecular Pathology Research Center, Peking Union Medical College Hospital, Chinese Academy of Medical Sciences and Peking Union Medical College, Beijing, 100730 China; 3 Department of Gynecologic Oncology, National Cancer Center, National Clinical Research Center for Cancer/Cancer Hospital, Chinese Academy of Medical Sciences and Peking Union Medical College, Beijing, 100021 China; 4 Geneplus-Beijing, Beijing, 102206 China; 5 National Institutes for Food and Drug Control, Beijing, 100050 China

**Keywords:** Epithelial ovarian cancer (EOC), Genomic methylation sequencing (GM-seq), Homologous recombination deficiency (HRD), Poly (ADP-ribose) polymerase inhibitors (PARPi)

## Abstract

**Background:**

The advent of poly (ADP-ribose) polymerase inhibitors (PARPi) over the past decade has significantly altered the management of epithelial ovarian cancer (EOC). We proposed that the etiology of homologous recombination deficiency (HRD) might underlie the variable responses to PARPi observed across patient populations.

**Methods:**

As part of the phase 2 study of the Chinese HRD Harmonization Project, we developed a genomic methylation sequencing (GM-seq) pipeline facilitated by the TET enzyme for the simultaneous identification of methylated modifications and genetic variations in EOC tumor samples, and compared with established DNA sequencing-based HRD assays.

**Results:**

Somatic mutation and HRD scores were confounded by low tumor purity in our cohort of 98 locally advanced/advanced EOC patients. In samples with tumor purity ≥ 30% (*n* = 45), the GM-seq pipeline showed high consistency with DNA sequencing-based HRD assay, identifying genetic variations in homologous recombination repair (HRR) genes and HRD score with 92.6% (25/27) and 97.1% (33/34) consistency respectively, in addition to conducting methylation profiling. Moreover, different underlying mechanisms of HRD were associated with varying degrees of PARPi efficacy, with *BRCA1/2* LOH group having the best efficacy (median PFS, undefined), followed by *BRCA1* methylation group (median PFS, 23.4 months), and those with unknown etiology of HRD having the worst efficacy (median PFS, 8.8 months, *p* < 0.001).

**Conclusion:**

Our findings underscore the importance of considering HRD etiology when evaluating PARPi efficacy in EOC patients. The GM-seq pipeline, represents a significant advancement in HRD detection, enabling more accurate predictions of PARPi response.

**Supplementary Information:**

The online version contains supplementary material available at 10.1186/s40364-025-00843-6.

## Background

Epithelial ovarian cancer (EOC) is the most lethal gynecologic malignancy, with a five-year survival rate of 50% [1]. Primary debulking surgery followed by platinum-based chemotherapy has become the standard of care for advanced EOC since the 1980s [2]. The emergence of poly (ADP-ribose) polymerase inhibitors (PARPis) over the past decade has led to a major change in the management of EOC [3]. Three (olaparib, niraparib and rucaparib) and four PARPis (olaparib, niraparib, fluzoparib and pamiparib) have been approved by the US Food and Drug Administration (FDA) and National Medical Products Administration of China (NMPA) in EOC, respectively, for the maintenance treatment of patients with EOC who exhibit complete or partial response to platinum-based chemotherapy [4–10]. The efficacy of EOC tumors to PARPis is significantly attributed to the homologous recombination deficiency (HRD), which is characterized by the inability to repair double-strand breaks in DNA through homologous recombination. In addition to germline and somatic mutations in homologous recombination genes, HRD can result from epigenetic silencing of homologous recombination genes and other indirect mechanisms that disrupt the normal activity of BRCA proteins [11–13]. Therefore, in the context of EOC management, evaluating HRD status is of the utmost importance.

HRD assays including “MyChoice^®^CDx” [14] and “FoundationOne^®^CDx” [4] have been approved by FDA to determine HRD status. However, current HRD assays have demonstrated controversial effects in predicting patient response to PARPi [15–18]. We speculated that different etiologies of HRD may result in different responses to PARPi. Besides, despite several recent studies [19–24], little is known about the real-world impact of HRD on the therapeutic effects of chemotherapy and PARPi in Chinese EOC patients.

Currently, the gold standard for base-level resolution and quantitative DNA methylation analysis is bisulfite-based sequencing and its derived methods [25, 26]. However, the harsh depyrimidination step of bisulfite-based sequencing would degrade most of the DNA, making simultaneous analysis of methylation and mutation impossible. The recently developed ten-eleven translocation (TET)-assisted pyridine borane sequencing (TAPS) directly detects 5-methylcytosine (5mC) and 5-hydroxymethylcytosine (5hmC) without affecting unmodified cytosines [27]. This bisulfite-free method is supposed to be much less destructive than bisulfite treatment. However, the performance of TAPS-based methylation analysis of mutation detection is largely unknown.

To fill the knowledge and application gap, we initiated the Chinese HRD Harmonization Project to understand the variables in HRD detection and promote the application of HRD in clinic [28]. In phase 2 of the project, in addition to establishing standards and standard datasets of HRD assay [29], we also developed a TET enzyme-mediated genomic methylation sequencing (GM-seq) pipeline [30] to simultaneously identify the methylated modification and genetic variations from tumor samples of EOC patients. With this novel technology, we were able to explore the etiology of HRD from both genomic and methylomic perspectives, and its association with the efficacy of PARPi.

## Methods

### Epithelial ovarian cancer participants

The study was part of the Chinese HRD Harmonization Project, which was approved by the Ethics Committee of the Cancer Hospital, Chinese Academy of Medical Sciences and Peking Union Medical College (project ID: 20/473–2669) [28, 29]. All the procedure conformed to the principles of the Helsinki Declaration. Informed Consent was waived. Medical records of epithelial ovarian cancer patients from Cancer Hospital and Peking Union Medical College Hospital were surveyed retrospectively. Eligibility criteria included being at least 18 years old at diagnosis; a histological/pathologic diagnosis of epithelial ovarian cancer; FIGO stage III, or IV; treated with PARPi in the maintenance or recurrent setting, the primary endpoint was PFS, defined as the time from the first dose of PARPi to disease progression or death, whichever occurred first. The cutoff date for assessing disease progression and survival of participants was December 4th, 2023. Formalin-fixed paraffin-embedded (FFPE) cancerous tissue samples from surgically resected specimens were collected and reviewed by two independent pathologists to determine the histological type and neoplastic cellularity.

### DNA extraction, targeted capture and next-generation sequencing analysis

Genomic DNA (gDNA) was extracted from FFPE samples using the ReliaPrep™ FFPE gDNA Miniprep System (Promega, Madison, WI, USA) according to the manufacturer’s instructions. DNA concentration was measured by Qubit™ dsDNA HS Assay Kit (Invitrogen, Carlsbad, CA, USA). The Agilent 2100 BioAnalyzer (Agilent Technologies, Santa Clara, CA, USA) was utilized to assess the size distribution of DNA. Briefly, at least 10 slides (4 μm in thickness, tissue area ≥ 50 mm²) of FFPE tissue samples were collected from each patient. It yielded 86-1903ng genomic DNA (median: 1069ng) for each sample. We defined the quality of DNA into A, B, C, D 4 categories according to the quantity and integrity of DNA, with A ranked as the best quality. In our cohort, 89/98 samples were ranked as A, 8 ranked as B and 1 sample ranked as C (P046, no enough DNA left for the GM-seq).

Sequencing libraries of gDNA were constructed with the KAPA DNA Library Preparation Kit (Kapa Biosystems, Wilmington, MA, USA) following the manufacturer’s protocol. Libraries were hybridized by custom-designed biotinylated oligonucleotide probes (1021 + HRD), which was designed to cover coding sequencing or hot exons of the 1021 cancer-associated genes (~ 1.5Mbp) that frequently mutate in solid tumors and one set of HRD-score probes (~ 150 Kbp) evenly covers the whole genome which aims to assess genomic instability on a global scale (Table S1). Next-generation sequencing (NGS) was performed using the Gene + seq2000 sequencer (Geneplus, Suzhou, China) with 2 × 101 bp paired-end reads [31, 32].

### Genomic data analysis

After removing adapters and low-quality reads by realSeq2 (https://www.biorxiv.org/content/10.1101/2023.05.16.539668v1), the clean reads were mapped to the human reference genome (hs37d5) using BWA-mem2. The Picard software MarkDuplicates (Broad Institute, Cambridge, MA, USA) was used for duplications removal. Somatic single nucleotide variants (SNVs) and small insertions and deletions (indels) were determined by realDcaller2 (Geneplus-Beijing, inhouse). Cnvkit was employed to detect copy number alterations (CNVs). Copy number alterations with copy number ≥ 2.8 were considered the potential amplification and were manually confirmed with a CAN plot. A self-developed algorithm NCsv2 (Geneplus-Beijing, inhouse) was used to identify structural variations (SVs). All final candidate variants were manually verified with the integrative genomics viewer browser [32]. Tumor mutational burden (TMB) was calculated as the number of all nonsynonymous mutations per megabase (Muts/Mb) of genome examined as described previously [33, 34].

### GM-seq assay

Whole genome methylation sequencing of genomic DNA was performed using a TET enzyme-based DNA methylation sequencing platform called GM-seq as described previously [30, 35]. Before library construction, sequences with CpG totally methylated (positive references) and CpG totally unmethylated (negative references) were mixed into the samples as controls. DNA methylation sequencing libraries were constructed using Hieff NGS^®^ Ultima Pro DNA Library Prep Kit for Illumina (Yeason, Shanghai, China), including end repair, dA tailing, adaptor ligation. Then, 5-methylcytosine (5mC) and 5-hydroxymethylcytosine (5hmC) were oxidized to 5-carboxycytosine (5caC) using the TET2 oxidase, and then converted to dihydrouracil (DHU) under the catalysis of the reducing agent (pyridine borane). DHU can be used as a PCR template and recognized by a DNA polymerase that recognizes U. Through PCR enrichment, 5mC was converted to T for whole genome sequencing, which was performed using Gene + seq2000 sequencer (Geneplus, Suzhou, China). Adaptor sequences and low-quality reads were filtered out from the raw sequencing data using fastp software (v0.19.5) [36]. Clean reads were mapped to the human reference genome (hg19) using Sentieon software (version 202010). The average sequencing depth in our study was 125.4 × and the quality control data for whole genome methylation sequencing was detailed in Table S2. The CpG sites in the promoter region of HRR genes with a total sequencing depth ≥ 10 were included to evaluate the methylation ratio of HRR gene promoter. The methylation ratio of the gene promoter was calculated as the number of methylated CpG reads divided by the sum of methylated and normal CpG reads.

### Analysis of HRD score and BRCA1/2 bi-allelic loss of function (BILOF)

An HRD score algorithm were developed to calculate a score for each of the three features: the loss of heterozygosity (LOH), telomeric allelic imbalance (TAI), and large-scale state transitions (LST) [29], and the overall HRD score was the sum of LOH [37], TAI [38], and LST [39] scores. The cutoff of 40 for the HRD score was identified with over 95% sensitivity to detect those *BRCA1/2* deficient EOC tumors [40], and verified in an independent cohort of PARPi treated patients (data not shown). *BRCA1/2* BILOF was defined as meeting one of the following two criteria: one allele has class 4/5 mutation and the other allele as LOH, or two class 4/5 mutations in either *BRCA1* or *BRCA2*.

### Statistical analyses

Data are presented as means ± standard deviation (SD) for continuous variables and as percentage for categorical variables. Differences between groups were tested by use of the Chi square or Fisher-exact tests for categorical variables, non-parametric test for non-normally distributed continuous variables and parametric for normally distributed continuous variables. PFS was defined as the time interval from the start of PARPi maintenance therapy to disease progression, with censoring of patients who are lost to follow-up. Survival analysis was undertaken using Kaplan–Meier methodology. All functions used belongs to R package stats (version 4.2.3). Two-tailed *P* < 0.05 was considered statistically significant.

## Results

### Patient and sample characteristics of EOC patients

We retrospectively surveyed the medical records of patients from two premier medical institutions in China: Peking Union Medical College Hospital (Beijing, China) and Cancer Hospital, Chinese Academy of Medical Sciences (Beijing, China) to recruit 98 ovarian cancer patients (Table 1) for the study. Among them, 94 (95.9%) patients were diagnosed with high-grade serous carcinoma, and 3 (3.1%) had endometrioid carcinomas. The majority of patients, 62 (63.3%), presented at stage Ⅲ, while 32 (32.6%) were at stage Ⅳ. Treatment regimens were as follows: olaparib was administered to 51 (52.0%) patients, niraparib was prescribed to 42 (42.9%) patients, and fluzoparib was given to 1 (1.0%) patient.

**Table 1:** Population characteristics (*N* = 98)

	*n* = 98
Histology, n (%)	
High-grade serous carcinoma	94 (95.9)
Endometrioid carcinomas	3 (3.1)
Unknown	1 (1.0)
Tumor stage, n (%)	
III	64 (65.3)
IV	34 (34.7)
PARP inhibitor, n (%)	
Olaparib	51 (52.0)
Niraparib	42 (42.9)
Fluzoparib	1 (1.0)
Unspecified	4 (4.1)
HRD status, n (%)	
Positive	62 (63.3)
Negative	36 (36.7)
BRCA altered, n (%)	
Germline mut	33 (33.7)
Somatic mut	13 (13.3)
CNL only	7 (7.1)
Wild type	45 (45.9)
Other HRR altered, n (%)	
Germline mut	5 (5.1)
Somatic mut	11 (11.2)
CNL only	19 (19.4)
Wild type	63 (64.3)
Neoplastic cellularity (pathology), %	
Median (min-max)	30 (1–95)
Tumor purity (ABSOLUTE), %	
Median (min-max)	39 (13–100)

Mut, mutation; CNL: copy number loss

Formalin-fixed paraffin-embedded (FFPE) tissue samples from these patients were retrieved for comprehensive genomic and methylomic analysis through NGS-based DNA sequencing and GM-seq. Neoplastic cellularity, as assessed by central pathology review, varied widely from 1% to 95% (median 30%, Table 1). Tumor purity, independently evaluated from DNA sequencing data using the ABSOLUTE algorithm [41], ranged from 13% to 100% (median 39%). A positive correlation was observed between neoplastic cellularity assessed by the pathologist and tumor purity evaluated by the bioinformatical algorithm (Figure S1). The median sequencing depth achieved was 1527 (1021-HRD panel, range: 471–2247) and 127.0 for GM-seq (range: 71.8–220.5).

Of the 98 patients, 62 (63.3%) were identified as HRD positive, defined as HRD scores ≥ 40 or a loss-of-function mutation in either *BRCA1* or *BRCA2*. Loss-of-function mutations in *BRCA1/2* were observed in 46 (47.0%) patients, including 33 (33.7%) with germline *BRCA1/2* mutations and 13 (13.3%) with somatic *BRCA1/2* mutations (Table 1). In addition, 7 (7.1%) patients had copy number loss (CNL) only in *BRCA1/2*. An additional 16 (16.3%) patients carried loss-of-function mutations in other homologous recombination repair (HRR) genes, including 5 (5.1%) with germline mutations and 11 (11.2%) with somatic mutations, and 19 (19.4%) patients carried other HRR CNL only (Table 1). Among other mutations observed across 1021 genes, *TP53* mutations occurred most frequently (79%), followed by *MYC* amplifications (27%). Other notable mutations included those in *NF1* (18%), *RAD21* (17%), *RAD51C* (12%), and *RECQL4* (11%). These findings underscore the genetic heterogeneity and complexity of ovarian cancer, highlighting the importance of comprehensive molecular profiling for effective treatment planning.

### Somatic mutation and HRD scores were confounded by low tumor purity

**Fig. 1:**
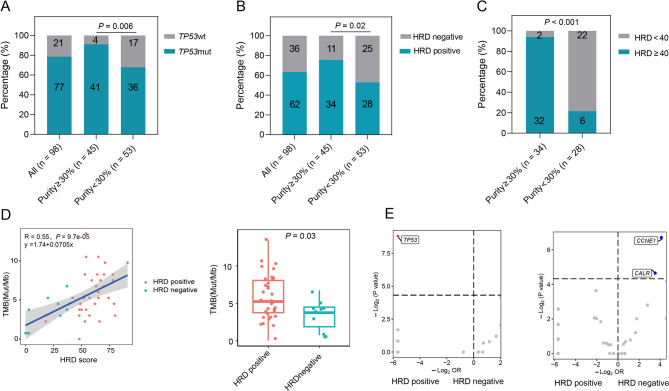
Correlation between HRD status and genomic features. **(A)** *TP53* mutation detection rose monotonically from 67.9% (*n* = 36/53) in the < 30% neoplastic cellularity bin to 91.1% (*n* = 41/45) in the ≥ 30% neoplastic cellularity, suggesting that the detection of *TP53* mutation is associated with neoplastic cellularity. **(B)** HRD positive detection rose monotonically from 52.8% (*n* = 28/53) in the < 30% neoplastic cellularity bin to 75.5% (*n* = 34/45) in the ≥ 30% neoplastic cellularity, suggesting that the detection of HRD positive is associated with neoplastic cellularity. **(C)** HRD score ≥ 40 detection rose monotonically from 21.4% (*n* = 6/28) in the below 30% neoplastic cellularity bin to 94.1% (*n* = 32/34) in the ≥ 30% neoplastic cellularity in HRD-positive group, suggesting that the detection of HRD positive is associated with neoplastic cellularity. **(D)** Pearson’s correlation was applied to analyse the correlation between TMB and HRD score. The regression line (blue) and 95% confidence band (shaded) are shown. And The box plot showed that the TMB level between HRD positive and negative group. **(E)** Enrichment analysis of somatic mutations between HRD-positive and HRD-negative groups. The x-axis represents the odds ratio (OR), and the y-axis represents the *p*-value. The horizontal dashed line indicates a *p*-value of 0.05, and the vertical dashed line indicates an OR of 1. HRD: Homologous recombination deficiency; TMB: tumor mutation burden

As approximately 96% of high-grade serous ovarian adenocarcinomas have somatic *TP53* mutations [42], we used the prevalence of *TP53* mutations to evaluate the impact of neoplastic cellularity on mutation detection (Fig. 1A). A disproportionate number of samples with *TP53* mutation were identified in the high neoplastic cellularity group (alterations in 41/45 high-purity versus 36/53 low-purity, *p* = 0.006). Moreover, there were more HRD-positive patients in the high neoplastic cellularity group (34/45 versus 28/53, *p* = 0.02, Fig. 1B) and more HRD-positive patients with HRD score ≥ 40 in the high neoplastic cellularity group (32/34 versus 6/28, *p* < 0.001, Fig. 1C). These findings further underscore that low neoplastic cellularity may obscure the analyses of mutational features and HRD score of the actual neoplastic cells [43, 44].

To overcome the tumor cellularity issue, further studies on HRD, mutation and methylation profiles were focused on the subset of tumors with higher tumor purity evaluated by ABSOLUTE (≥ 30%, *n* = 45).

In spite of the general low tumor mutation burden (TMB) in ovarian patients, TMB was positively correlated with HRD score, and higher TMB was found in HRD-positive group (*R* = 0.55, 5.25 versus 3.75, *p* = 0.03, Fig. 1D). We further analyzed genetic alterations enrichment in HRD-positive or -negative patients. A higher percentage of *TP53* mutation was identified in the HRD-positive group, and a higher percentage of *CCNE1* and *CALR* copy number gains were noticed in the HRD-negative group (Fig. 1E).

### Consistency of HRR mutation and HRD score between 1021-HRD and GM-Seq

We next tested the performance of GM-Seq in the concurrent analysis of methylation and genetic variation by comparing with the 1021-HRD panel DNA sequencing.

**Fig. 2:**
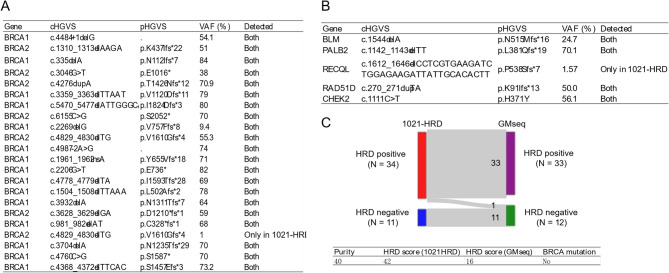
Consistency of HRR mutation detection between 1021-HRD and GMseq. **(A)** Concordance between 1021-HRD and GMseq for *BRCA1/2* mutation detection in samples with ≥ 30% neoplastic cellularity. **(B)** Concordance between 1021-HRD and GMseq for detection of other HRR gene mutations in samples with ≥ 30% neoplastic cellularity. **(C)** Concordance between 1021-HRD and GMseq for HRD status in samples with ≥ 30% neoplastic cellularity (*n* = 45). HRR: Homologous recombination repair; HRD: Homologous recombination deficiency; GMseq: genomic methylation sequencing

Among 22 *BRCA1/2* mutations, including somatic or germline, single nucleotide variant (SNV) or small insertion or deletion (InDel) identified by 1021-HRD, 21 (95.5%) of them were detected in the GM-Seq results as well (Fig. 2A), except for the low-frequency *BRCA2* p.V1610Gfs*4 mutation (variant allele frequency [VAF] = 1.00%) (Figure S2A). In terms of the 5 HRR mutations, only *RECQL* p.P538Sfs*7 mutation (VAF = 1.57%) was missed in the GM-Seq (Fig. 2B and S2B). We speculated the intermediate length of deletion (35 bp) and the low VAF of this mutation were the major reasons.

Among 34 HRD-positive samples identified by 1021-HRD, only one was determined as negative by GM-seq. The inconsistent sample was BRCA wild-type, with HRD score at 42 in the 1021-HRD assay and 16 in the GM-Seq assay (Fig. 2C). Therefore, 1021-HRD panel and GM-Seq demonstrated an extremely high concordance in detecting mutations and identifying HRD.

### Different cause of HRD was associated with papri efficacy

Bi-allelic alterations in HRR genes are necessary for HRD according to the two-hit hypothesis. We tested how the bi-allelic loss of function (BILOF) of HRR genes happened and led to genomic scarring in 34 tumors with positive HRD and higher neoplastic cellularity [45].

**Fig. 3:**
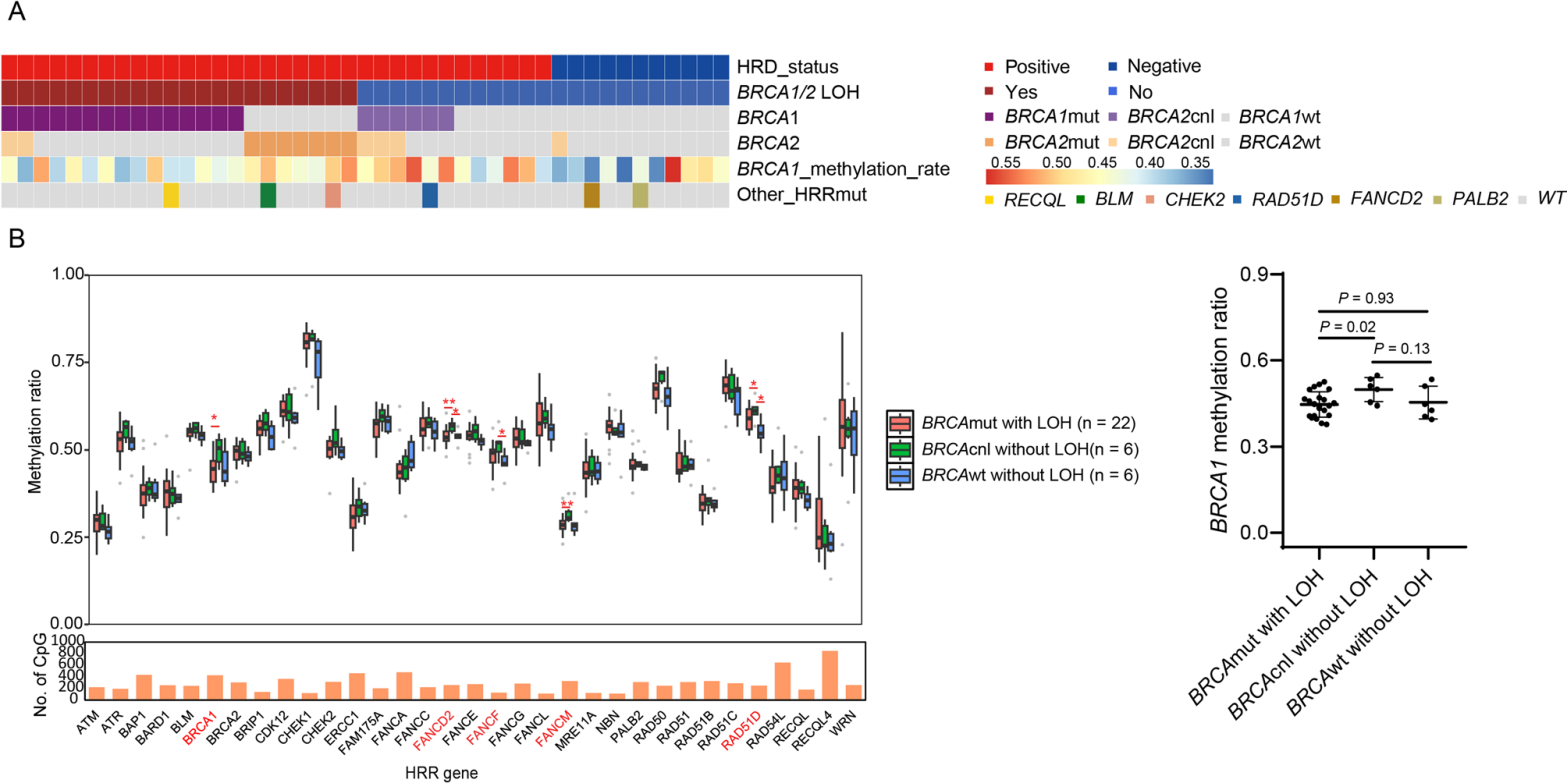
HRD positive underlying mechanisms. The heatmap reveals the The most common causes of HRD in the samples with ≥ 30% neoplastic cellularity (*n* = 45). **(B)** Samples were stratified into three groups: *BRCA*mut with LOH, *BRCA*cnl without LOH, and *BRCA*wt without LOH. Box-and-whisker plots show HRR gene methylation levels among the three groups. HRR: Homologous recombination repair; HRD: Homologous recombination deficiency. HRR: Homologous recombination repair; HRD: Homologous recombination deficiency. **(C)** The scatter plot shows the methylation level of *BRCA1* promoter between *BRCA*mut with LOH, *BRCA*cnl without LOH, and *BRCA*wt without LOH group

For the 15 *BRCA1* mutated and 7 *BRCA2* mutated samples, loss of heterozygosity (LOH) of the wildtype allele was observed and accounts for the “second-hits” occurring in BRCA-related tumors (*BRCA1/2* LOH group). Among the 22 *BRCA1/2* LOH patients, two of them (P051, P087) had *BRCA2* copy number loss (CNL) in addition to *BRCA1* LOH (Fig. 3A). For the 6 HRD-positive patients with *BRCA1* somatic CNL, 3 (P020, P049, P070) had concurrent *BRCA2* somatic CNL (Fig. 3A, *BRCA1/2* CNL group). Other HRR mutations occurred either with *BRCA1/2* LOH (*BLM*,* FANCM*,* RECQL*) or with *BRCA1* somatic CNL samples (*RAD51D*) in the HRD-positive group, thus there were 6 HRD-positive patients without definite HRR loss of function mutation, and we defined this group as HRD-positive with an unknown etiology.

We then compare the promoter methylation level of HRR gene among these three groups. Increased methylation level at the promoters of *BRCA1*, *FANCD2*, *FANCM* and *RAD51D* was found in the *BRCA1/2* CNL group compared to *BRCA1/2* LOH group, and increased methylation level at the promoters of *FANCD2*, *FANCF* and *RAD51D* was found in the *BRCA1/2* CNL group compared to HRD-positive group with an unknown etiology (Fig. 3B). Interestingly, patient P086 in the HRD-positive group with an unknown etiology had both *RAD51D* CNL and increased methylation on *RAD51D*, which may contribute to the high HRD score in this patient.

**Fig. 4:**
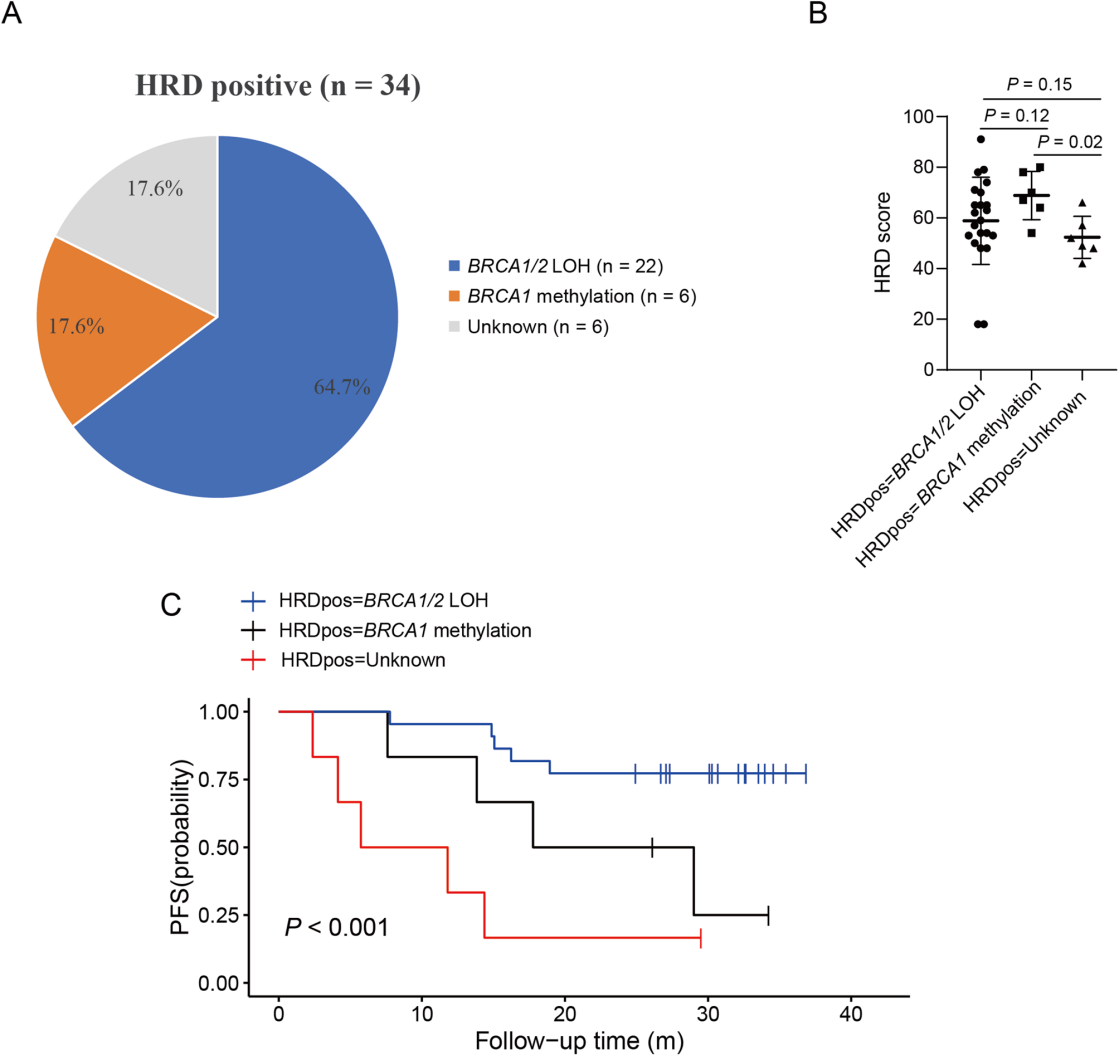
Underlying mechanisms of HRD was associated with PARPi efficacy. **(A)**The pie chart shows the distribution of different mechanisms of HRD positive. *BRCA1/2* LOH accounted for 64.7% (22/34) of cases, *BRCA1* methylation for 17.6% (6/34), and unknown mechanisms for 17.6% (6/34). **(B)** The scatter plot shows the HRD score levels among the three groups: *BRCA1/2* LOH, *BRCA1* methylation, and unknown. **(C)** Kaplan–Meier curves were constructed for patients with *BRCA1/2* LOH (median PFS, undefined), *BRCA1* methylation (median PFS, 23.4 months) and those with unknown (median PFS, 8.8 months). HRD: Homologous recombination deficiency; PFS: progression-free survival

As the 6 patients with *BRCA1/2* CNL had higher methylation level of *BRCA1* promoter than HRD-positive patients with other etiologies (medium methylation ratio of *BRCA1* promoter: 0.51 versus 0.45, *p* = 0.03; Fig. 3C), we proposed that high methylation level of *BRCA1* promoter combined with *BRCA1* CNL contributed to the etiology of HRD of these patients and defined them as *BRCA1* methylation group (Fig. 4A). The HRD score was comparable between *BRCA1/2* LOH group and *BRCA1* methylation group, however, the HRD score was lower in the etiology-unknown group (Fig. 4B).

Among the 34 patients with positive HRD and tumor purity ≥ 30%, all had high-grade serous carcinoma and received PARPi as first-line maintenance therapy, with 67.6% (23/34) having stage III disease. Survival analysis showed that patients with *BRCA1* methylation had longer PFS with PARPi maintenance therapy than the patients with unknown etiology, but shorter PFS than patients with *BRCA1/2* LOH (medium PFS: 23.4 m versus 8.8 m versus undefined, *p* < 0.001, Fig. 4C). This survival difference was observed as well in the stage III and IV subgroups (Figure S3).

## Discussion

We present a combined genomic and methylome analysis of 98 epithelial ovarian cancer specimens that exhibit a range of neoplastic cellularity representative of the clinicopathologic spectrum of this disease. We demonstrated that low cellularity could obscure the analyses of mutation and HRD score of the actual neoplastic cells, thus highlighting the importance of considering neoplastic cellularity when analyzing HRD and mutations. We also demonstrated the GM-seq pipeline could detect HRR mutations and HRD score with high consistency to DNA-based NGS sequencing in addition to the its analysis of methylated modification. With this novel technology, we explored the cause of HRD and found patients with *BRCA1* methylation-induced HRD had better efficacy of PARPi than those with unknown cause of HRD, but worse efficacy of PARPi than that of *BRCA1/2* LOH.

Next-generation sequencing has been widely used in clinical practice. A prominent problem in the analysis of NGS data is to deconvolve the mixture to identify the reads associated with tumor cells as the reads obtained from NGS of tumor samples often consist of a mixture of normal and tumor cells, which can be of multiple clonal types. Most cancer NGS assays are validated to detect somatic mutations at variant allele fraction of as low as 5–10% and generally require tissue specimens containing at least 20% tumor nuclei [46]. However, in the analysis of HRD, we found higher requirement for tumor purity (≥ 30%), as quantitative somatic copy number variation analysis is necessary in the analysis of HRD, which calculated genomic-scar-based HRD score with the unweighted sum of LST, TAI, and LOH events [40].

An integrative genomic analysis of cases with and without HRD revealed that the likeliest etiology for HRD in the vast majority of cases is bi-allelic inactivation of bona fide HR genes [47]. It was reported that bi-allelic inactivation of HR genes was found to identify almost 90% of cases with a functional HR defect [48]. In our study, we identified two tumors (P004, P031) with either a bi-allelic *BRCA1* or *BRCA2* mutation without evidence of a functional deficit in HR. These two cases did not display evidence of intra-genic deletions or reversion mutations in the tumor. One of the patients (P031) had a somatic mutation of *BRCA1* at the VAF of 9.4% at the tumor purity of 35%. We speculated that dysregulation of the HR pathway may have occurred late in tumor evolution in this particular patient, hence not leaving a mark on the genome. This phenomenon was observed in BRCAness associated breast cancer as well [48]. As to the patient with germline *BRCA2* mutation (P004), we could not exclude the possibility of alteration in other proteins such as *TP53BP1*,* RIF1*,* HELB*,* PTIP* or *MAD2L2* may restore DNA repair in this *BRCA2* deficient tumor cells [49–53].

In ovarian cancer, *BRCA1* promoter methylation occurs in 10–20% of cases and is mutually exclusive of *BRCA1* mutation [54]. However, in breast cancer, there was not a clear role for aberrant HR gene expression or *BRCA1* promoter methylation in mediating functional HR deficiency, although methylation of BRCA1 is enriched in breast cancers compared to normal breast epithelium [48]. In our cohort, all the 6 patients with *BRCA1* methylation as the second-hit was with *BRCA1* copy number loss but not loss of function mutation (Fig. 3A). We still had 6 patients with dysfunctional HR who did not have a bi-allelic alteration in *BRCA1* or *BRCA2* gene either with LOH or *BRCA1* methylation. Other causes such as transcriptomic changes or protein changes in HR genes may also be associated with functional HRD in epithelial ovarian cancer.

The different responses to PARPi of the 3 groups of HRD patients (BRCA LOH, BRCA1 methylation, and cause unknown) was somehow consistent with previous studies that generally BRCAness associated HRD patients respond better than BRCA-wild-type HRD patients to PARPi [4, 8, 14, 17]. In terms of *BRCA1* promoter hypermethylation, the clinical outcome of EOC patients with *BRCA1* promoter hypermethylation has been compared to patients with germline *BRCA1* mutations and those with wild-type BRCA [11]. Survival of patients with methylated *BRCA1* promoters (*n* = 11, 35.6 months) was significantly shorter than that of both patients with wild-type *BRCA1* (*n* = 30, 63.3 months) and *BRCA1* mutations (*n* = 22, 78.6 months) [55]. In a recently published study, *BRCA1* Promoter Methylation was associated with poor prognosis [56]. This led to the hypothesis that *BRCA1* promoter hypermethylation may actually be a marker of aggressive disease. Though patients in these studies were not treated with PARPi, the poor prognosis compared with B*RCA1/2* mutant patients was consistent with our results. Considering current genomic-scar-based HRD score applied in clinical trials and its controversial effects in predicting patient response to PARPi and other antiangiogenic therapy [15–18], the European Society for Medical Oncology (ESMO) has highlighted that better assays are needed to identify HRD patients for first-line treatment of ovarian cancer [57]. We proposed that larger cohorts that explore the cause of phenotypic deficiencies in HR may be one of the potential approaches.

Genetic alterations affecting HR pathway-related genes other than *BRCA1/2* have been linked to response to cancer predisposition or HR-targeted therapies in multiple other cancers [58–63]. In our study, patient P086 who had both *RAD51D* CNL and increased methylation on *RAD51D*, this bi-allele alteration may contribute to the high HRD score in this patient. Four LOF mutations in HRR genes (*BLM*,* FANCM*,* RAD51D*,* RECQL*) were identified in concurrent with *BRCA1/2* bi-allele alteration. However, the other 2 LOF mutations in HRR genes (*FANCD2*,* PALB2*) did not result in HRD. This was consistent with the notion that bi-allelic germline and/or somatic alterations in HR genes, rather than the mere presence of a mutation in these genes, lead to phenotypic functional defects in HR.

There were some limitations in this study. Firstly, this study was a retrospective study and only patients treated with PAPRi were included, there were high rate of germline *BRCA1/2* mutant patients, which was not representative of the whole EOC population, the potential bias added the complexity to data interpretation. Secondly, the sample size of patients for analyzing the efficacy of PRAPi was relatively small because we blindly selected many samples with low tumor cellularity. Thus, the small sample size may limit the statistical power of our findings and need to be verified by large prospective studies. Moreover, in vivo validation such as patient-derived xenograft (PDX) models is warranted in future prospective studies.

## Conclusions

In conclusion, we demonstrated low tumor cellularity could obscure the analyses of mutation and HRD score of the actual neoplastic cells. Our work also highlights the importance of having bi-allelic alterations in the HR pathway, as opposed to ‘single-hits’ to result in a functional deficiency in HR. We extend the significance of a comprehensive genetic assessment of the HR pathway genes as well as the underlying cause of HRD to the treatment choice for epithelial ovarian cancer patients. In a translational setting, our GM-seq pipeline can allow for a more thorough interpretation of genomic alterations and cause of HRD simultaneously and warrants further investigation in large cohorts from prospective clinical trials.

## Supplementary Information

Below is the link to the electronic supplementary material.

**Supplementary Material 1**: **Figure S1**. Correlation between neoplastic cellularity assessed by the pathologist and tumor purity valuated by the bioinformatical algorithm.

**Supplementary Material 2**: **Figure S2**. Two low frequency mutations missed in GMseq. (A) The upper graph shows the low frequency *BRCA2* mutation reads in 1021-HRD and the lower graph shows missing of *BRCA2* mutation read in GMseq. (B) The upper graph shows the low frequency RECQL mutation reads in 1021-HRD and the lower graph shows the low quality RECQL mutation reads in GMseq.

**Supplementary Material 3**: **Figure S3**. The probability of PFS in stage III or IV HRD-positive patients with different etiologies.

**Supplementary Material 4**: **Table S1**. List of 1021 cancer-related genes.

**Supplementary Material 5**: **Table S2**. The quality control data of GMseq.

## Data Availability

The data generated in this study were deposited in the National Genomics Data Central (NGDC) database under accession number HRA011690 and data were available upon reasonable request to the corresponding author.

## Abbreviations

PARPi: Poly (ADP-ribose) polymerase inhibitors
EOC: Epithelial ovarian cancer
GM-seq: Genomic methylation sequencing
HRR: Homologous recombination repair
FDA: Food and Drug Administration
NMPA: National Medical Products Administration of China
HRD: Homologous recombination deficiency
TET: Ten-eleven translocation
TAPS: Ten-eleven translocation (TET)-assisted pyridine borane sequencing
5mC: 5-methylcytosine
5hmC: 5-hydroxymethylcytosine
FFPE: Formalin-fixed paraffin-embedded
NGS: Next-generation sequencing
SNVs: Single nucleotide variants
Indels: Small insertions and deletions
CNVs: Copy number alterations
SVs: Structural variations
TMB: Tumor mutational burden
DHU: Dihydrouracil
BILOF: Bi-allelic loss of function
LOH: Loss of heterozygosity
TAI: Telomeric allelic imbalance
LST: Large-scale state transitions
SD: Standard deviation
CNL: Copy number loss
ESMO: European Society for Medical Oncology
