# Supplementary figures and images for: Comprehensive assessment of homologous recombination deficiency via simultaneous methylation and mutation analysis in epithelial ovarian cancer: implications for PARP inhibitors efficacy
Source: Biomark Res. 2025 Oct 10;13:123. doi: 10.1186/s40364-025-00843-6 (PMC12512315; doi:10.1186/s40364-025-00843-6)

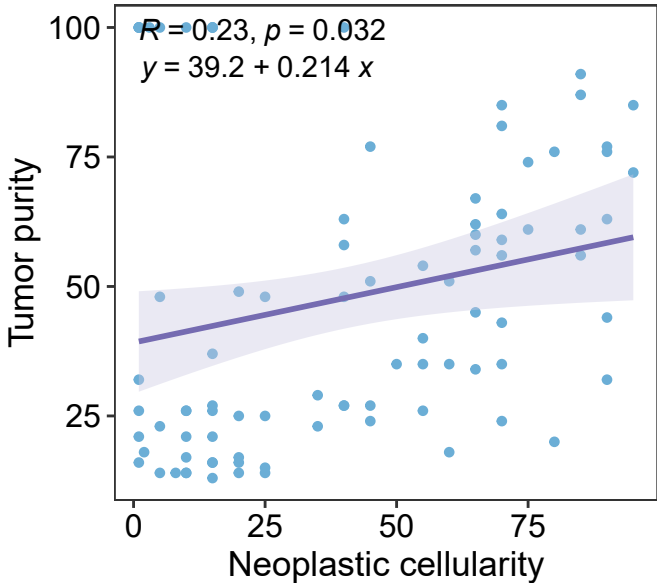

Supplement: Supplementary file 1 — Supplementary Material 1: Figure S1. Correlation between neoplastic cellularity assessed by the pathologist and tumor purity valuated by the bioinformatical algorithm. [file 40364_2025_843_MOESM1_ESM.pdf]

A

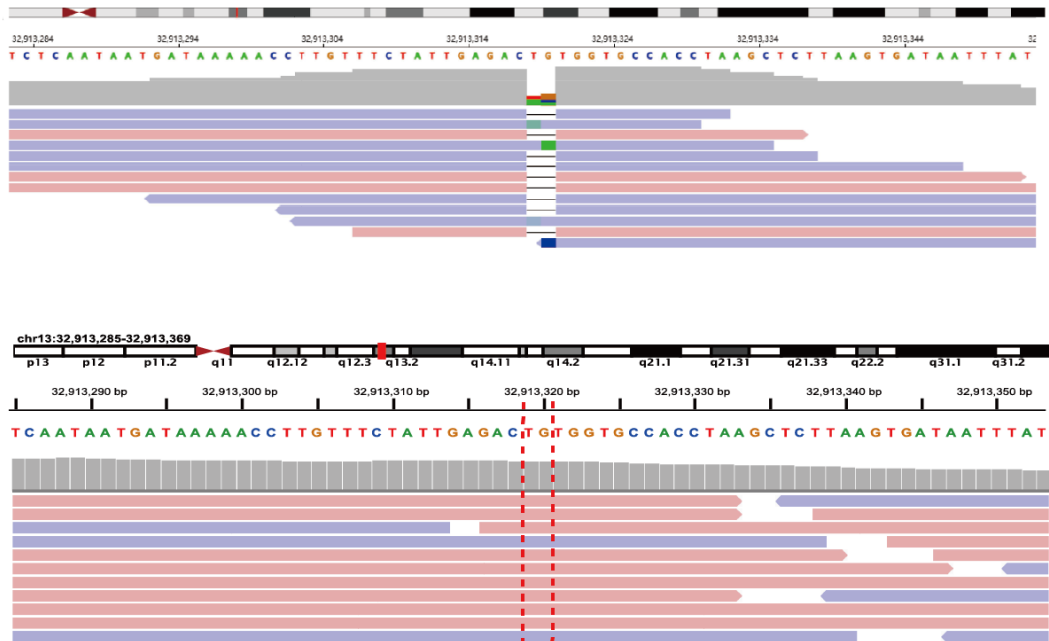

B

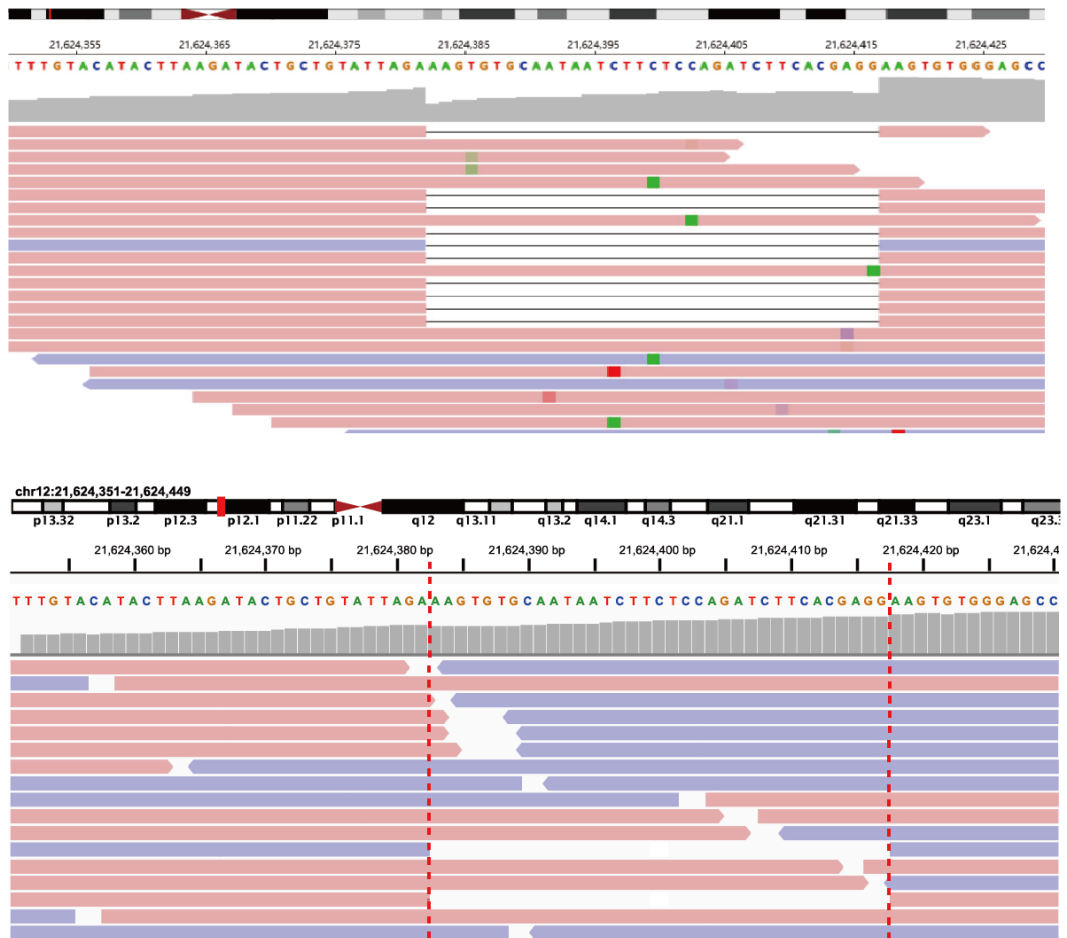

Supplement: Supplementary file 2 — Supplementary Material 2: Figure S2. Two low frequency mutations missed in GMseq. (A) The upper graph shows the low frequency BRCA2 mutation reads in 1021-HRD and the lower graph shows missing of BRCA2 mutation read in GMseq. (B) The upper graph shows the low frequency RECQL mutation reads in 1021-HRD and the lower graph shows the low quality RECQL mutation reads in GMseq. [file 40364_2025_843_MOESM2_ESM.pdf]
