# Supplementary material for: Comprehensive assessment of homologous recombination deficiency via simultaneous methylation and mutation analysis in epithelial ovarian cancer: implications for PARP inhibitors efficacy
Source: Biomark Res. 2025 Oct 10;13:123. doi: 10.1186/s40364-025-00843-6 (PMC12512315; doi:10.1186/s40364-025-00843-6)
Supplement: Supplementary file 3 — Supplementary Material 3: Figure S3. The probability of PFS in stage III or IV HRD-positive patients with different etiologies. [file 40364_2025_843_MOESM3_ESM.pdf]

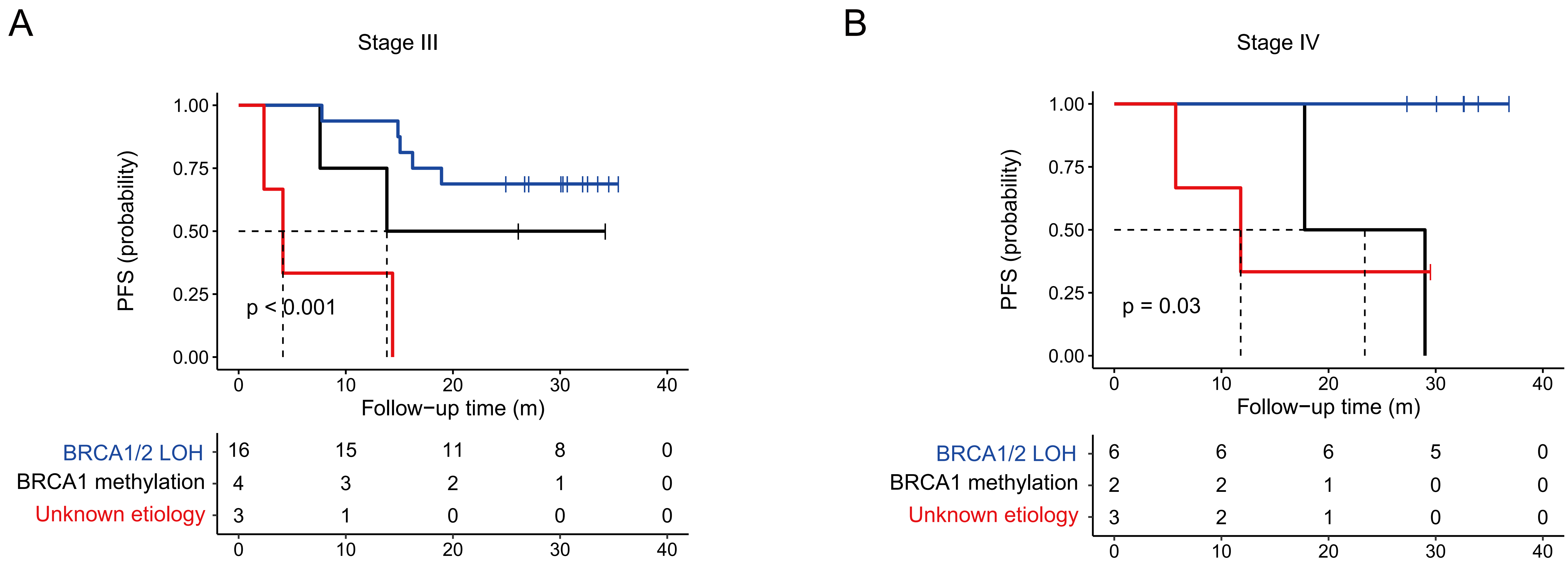

A
Stage III
PFS (probability)
1.00
0.75
0.50
0.25
0.00
p < 0.001
0
10
20
30
40
Follow−up time (m)
BRCA1/2 LOH 16 15 11 8 0
BRCA1 methylation 4 3 2 1 0
Unknown etiology 3 1 0 0 0
B
Stage IV
p = 0.03
BRCA1/2 LOH 6 6 6 5 0
BRCA1 methylation 2 2 1 0 0
Unknown etiology 3 2 1 0 0
